# Spondylitis with epidural abscess and spinal tuberculoma as a major cause of myeloradiculopathy: a case report

**DOI:** 10.1016/j.radcr.2022.10.073

**Published:** 2022-11-25

**Authors:** Eppy Buchori, Puspita Permata Sari

**Affiliations:** Department of Radiology, Faculty of Medicine, Universitas Padjadjaran, Hasan Sadikin General Hospital, Jl. Pasteur No. 38 Sukajadi, Bandung, Indonesia

**Keywords:** Tuberculosis, TB, Spinal tuberculosis, Tuberculoma, Epidural abscess, Chest X-ray, MRI

## Abstract

Tuberculosis is caused by Mycobacterium tuberculosis, a slow-growing aerobic bacillus. The primary site of infections can be in the lungs, mediastinum lymph nodes, mesentery, gastrointestinal tract, genitourinary system, or any other viscera. We described a case of a 52-year-old Indonesian female who presented weakness in upper and lower extremities with low back pain and stiffness of the muscle since 4 months ago and getting worse over 1 week. She has no history of pulmonary tuberculosis. Then the patient underwent a laboratory examination, chest X-ray, vertebrae X-ray, and MRI examination for further evaluation. Both examinations revealed tuberculous spondylitis with epidural abscess and intramedullary tuberculoma. The patient was administered an antituberculosis drug therapy, and the surgery was scheduled after three months of treatment.

## Introduction

Tuberculosis (TB) is caused by the Mycobacterium tuberculosis complex, which has around 60 species. Only Mycobacterium tuberculosis (the most common), Mycobacterium bovis, Mycobacterium microti, and Mycobacterium africanum are known to affect humans [1]. The primary site of infections can be in the lungs, mediastinum lymph nodes, mesentery, gastrointestinal tract, genitourinary system, or any other viscera. The bacilli remain dormant for prolonged periods and multiply every 15 to 20 hours in aerobic conditions whenever favorable. Spinal infection is always secondary and is caused by hematogenous dissemination of the bacillus from a primary focus [1,2]. Some factors which increase the risk of becoming infected with tuberculosis are poverty, overpopulation, illiteracy, malnutrition, alcoholism, drug addiction, diabetes, immunosuppressive therapy, and HIV infection [1]. The disease affects people regardless of sex and age. The highest percentage of cases reported in 2018 was observed in men older than 15 years (57%), women (32%), and children below 15 years of age (11%). A total of 8.6% of tuberculosis cases wereobserved in individuals infected with HIV. It is estimated that the risk of developing tuberculosis is 20 to 37-fold higher in HIV-infected individuals compared to HIV-negative ones. The majority of cases were reported in Southeast Asia (44%), Africa (24%), and the Western Pacific (18%). The following eight countries are responsible for 2/3 of the global incidence: India (27%), China (9%), Indonesia (8%), the Philippines (6%), Pakistan (6%), Nigeria (4%), Bangladesh (4%), and South Africa (3%) [3].

## Case report

**Fig. 1:**
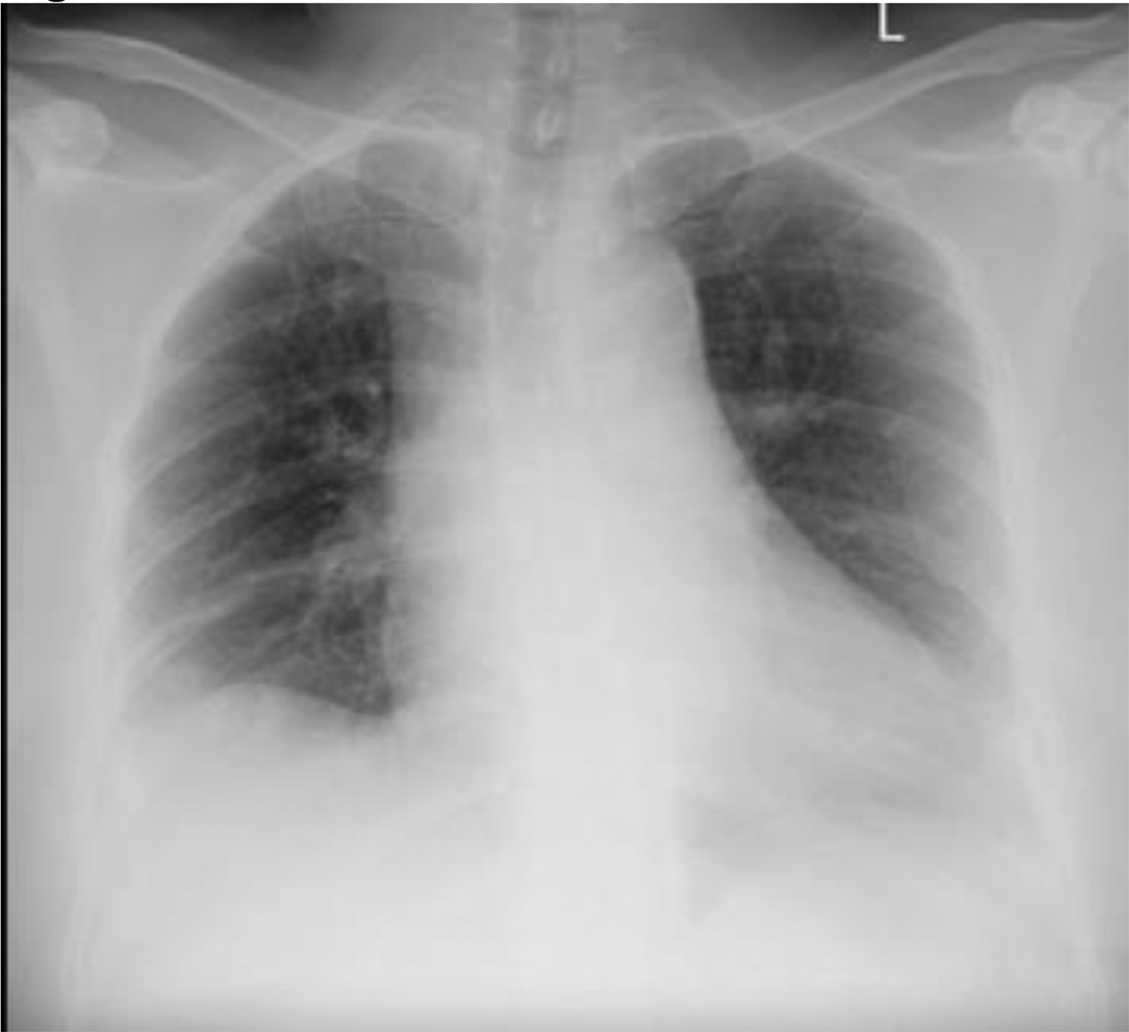
Chest X-ray showed cardiomegaly with atherosclerosis aorta, with no lung abnormalities.

**Fig. 2:**
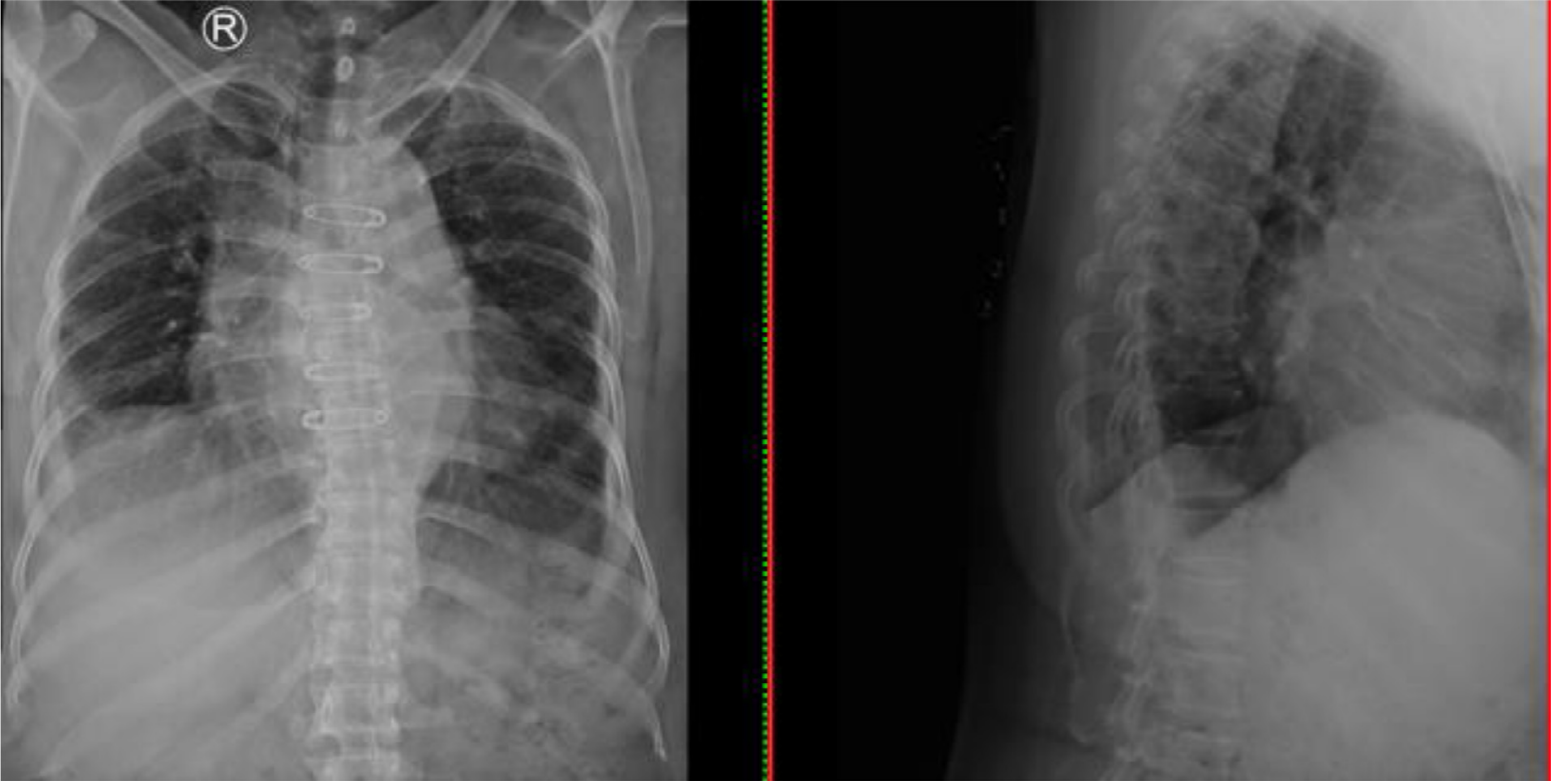
Compression fracture at the vertebrae thoracalis level 6. (a). Anteroposterior view. (b). Lateral view.

A 52-year-old Indonesian female complained about being weak in her upper and lower extremities with low back pain and muscle stiffness since four months ago and getting worse over the last week. She has no bladder or bowel dysfunction nor a history of pulmonary tuberculosis. Laboratory examinations diagnosed leukocytosis. Chest X-ray showed cardiomegaly with atherosclerosis aorta and no lung abnormalities (Fig. 1). Thoracolumbar X-rays obtained compression fracture at the vertebrae thoracalis 6 level (Fig. 2).

**Fig. 3:**
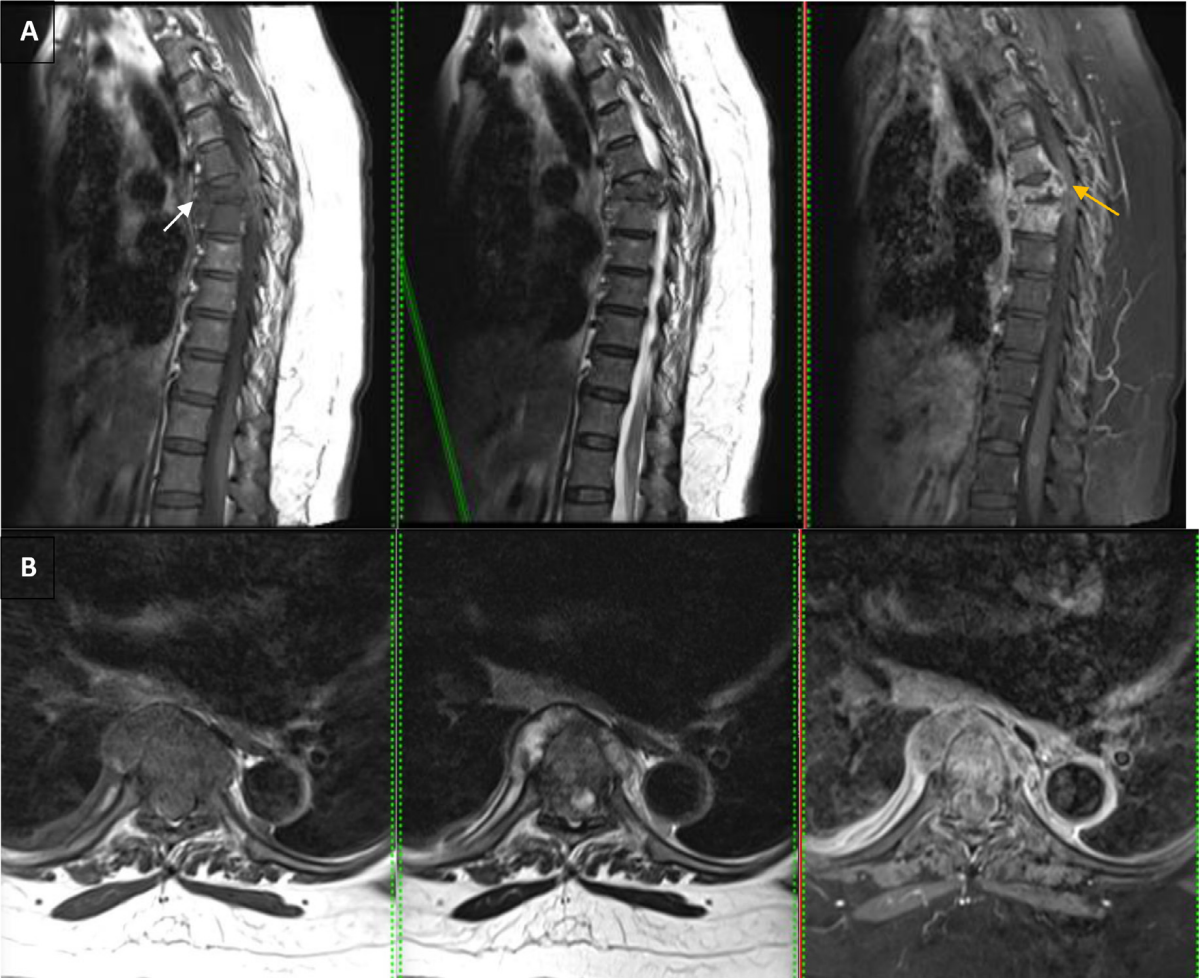

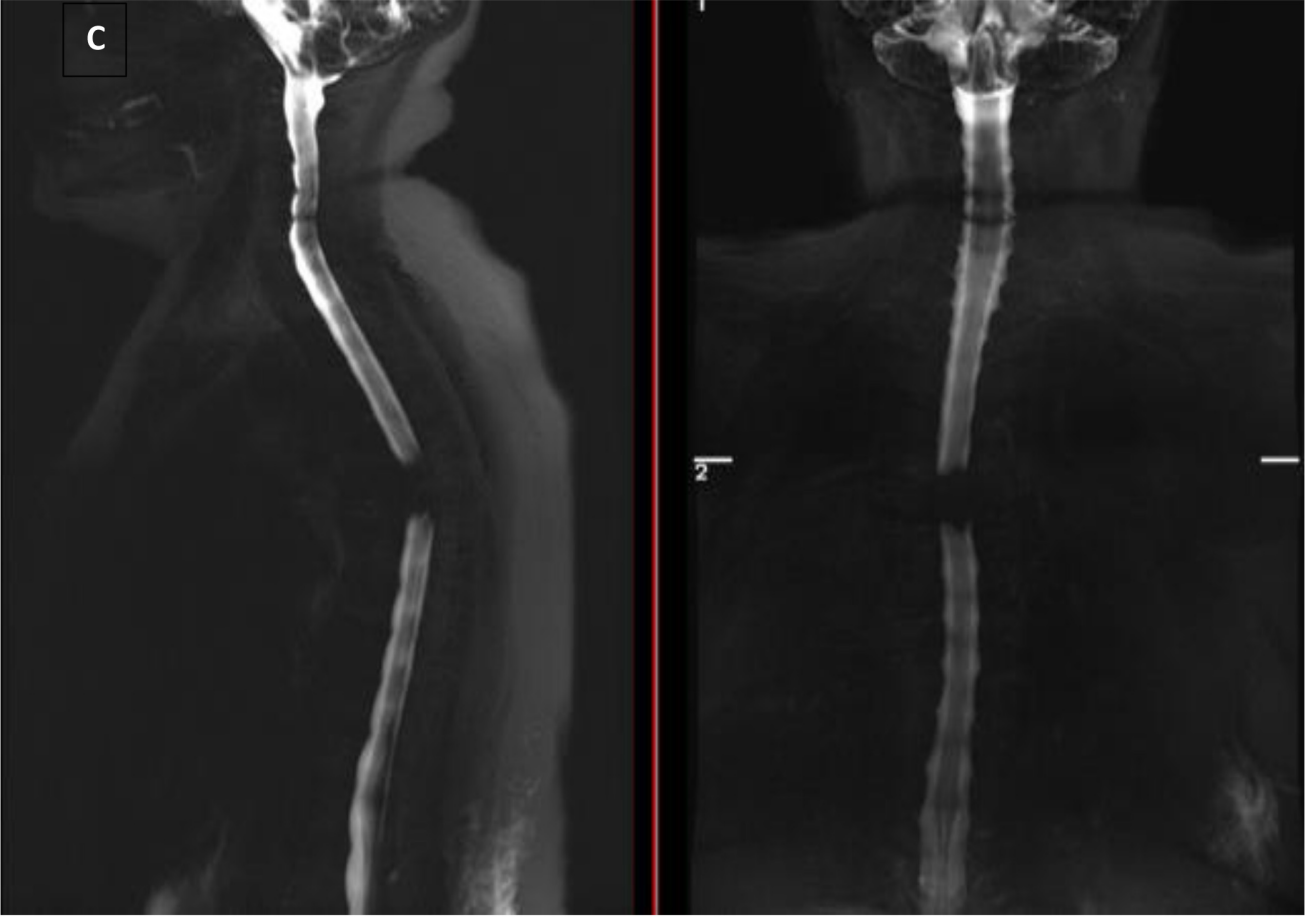
(a) Compression fracture thoracic 6 (white arrow), with bone marrow replacement, and epidural abscess (yellow arrow) after IV contrast administration on sagittal view. (b) Axial view (c). Sagital and coronal myelography showed stenosis.

A thoracolumbar MRI examination using intravenous (IV) contrast administration displayed compression fracture thoracic 6, bone marrow replacement of multiple upper thoracic vertebrae with an epidural abscess at thoracal level 6-7, which gives hypointense signals on T1-weighted and hyperintense signals on T2-weighted and enhancement at the margin. The lesion causes canal stenosis with a posteriorly displaced position and gives isointense signals on T1-weighted and hyperintense signals on T2-weighted in the spinal cord region at the level of that area (Fig. 3).

**Fig. 4:**
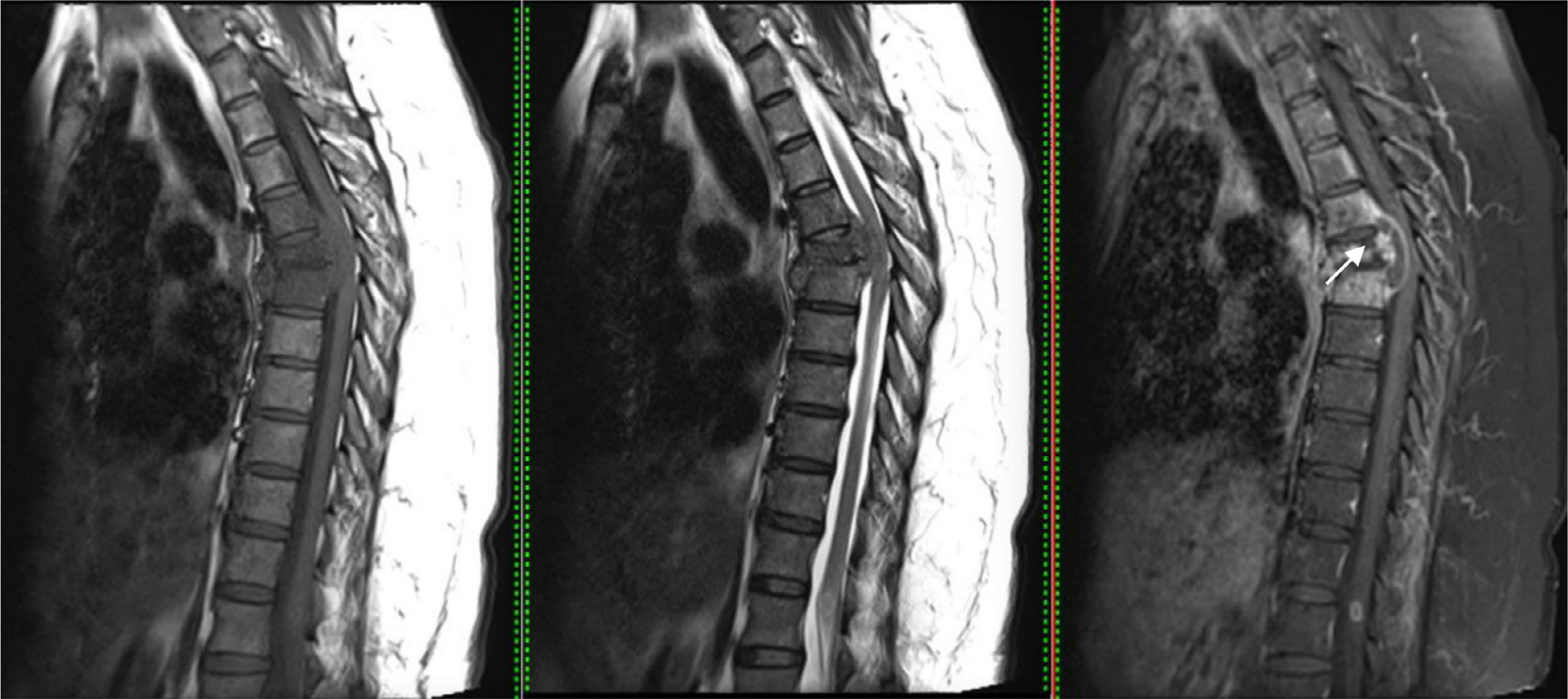
Ring shape with enhancement in the intramedullary lesion suggestive of spinal tuberculoma accidental findings on the sagittal view (white arrow).

However, T2-weighted of the lumbar spine detected an isointense signal at lumbar 1. There were isointense signals on the T1-weighted with a ring shape enhancement of the intramedullary lesion at the L1 after contrast administration (Fig. 4).

Accordingly, the patient was diagnosed with tuberculous spondylitis, epidural abscess, and intramedullary tuberculoma. The presentation of epidural abscess confirmed that surgery was required [4]. From these results, she was administered an antituberculosis drug therapy, and the surgery will be carried out after 3 months of treatment.

## Discussion

Tuberculosis is caused by Mycobacterium tuberculosis; it is a slow-growing aerobic bacillus. The primary site of infections can be in the lungs, mediastinum lymph nodes, mesentery, gastrointestinal tract, genitourinary system, or any other viscera. The bacilli remain dormant for prolonged periods and multiply every 15 to 20 hours in aerobic conditions whenever favorable. Spinal infection is always secondary and is caused by hematogenous dissemination of the bacillus from a primary focus [1].

Tuberculous spondylitis is extrapulmonary tuberculosis that requires early detection to prevent further neurological complications. The most common site for TB spondylitis is the thoracolumbar vertebra, followed by the thoracic vertebrae, lumbar vertebrae, and cervical vertebrae. Garg and Somvanshi [5] examined 30 cases of spinal tuberculosis and found 2 cases in the cervical part of the vertebra, 4 cases in the thoracic vertebrae, 21 cases in the lumbar vertebrae, and 3 cases in the sacral part of the vertebrae. For this particular patient, the location of the lesion was in the thoracal vertebral segment, while the infection was in the lumbar 1.

Spinal tuberculosis is a destructive form of tuberculosis. Generally, bone TB is seen from the destruction of the space of the intervertebral discs and adjacent corpus vertebrae. If it is already an advanced stage, it can cause a collapse of the spinal element and an anterior wedge that causes the formation of kyphosis and gibbus [5]. Skoura et al. [6] examined infections starting in the subchondral bone that spread slowly in the lower back and lumbar spine so that serious complications such as spinal collapse, spinal compression, and spinal deformities can occur. In the removed case, the patient has not yet reached the collapse stage, so it does not appear gibbus or kyphosis.

Generally, the main complaints of most patients (90%) in the early stages are symptoms of back pain, weight loss, loss of appetite, and fever. Some also experience neck pain, weakness of the lower extremities, upper jaw, weakened limbs, and deformities [7]. The main complaints (83%-100% of cases) are back pain caused by disruption of the intervertebral disc and spinal instability, nerve compression, or pathological fracture, exacerbated by spinal movement, coughing, and weight-bearing [8,9]. This situation of back pain may be caused by Mycobacterium tuberculosis, dormant in the vertebrae during primary infection. Primary focus can be active or dormant, apparent or latent, either pulmonary or extrapulmonary. Typical pulmonary TB symptoms and medical history supporting a radiological diagnosis (Primary Tuberculosis) are not primary symptoms. Kusmiati and Narendrani [10] stated that only 50% of bone and joint tuberculosis patients obtained TB images in their thoracic photo radiographs. On this patient, no infection on her chest X-ray was found.

Tuberculosis bacilli can travel from the lungs to the spine via Batson's paravertebral venous plexus or lymphatic drainage to the para-aortic lymph nodes. Patients with TB spondylitis also have complications of neurological deficits. This patient had a neurological deficit in which the left leg decreased motoric strength. This condition was caused by compression of the paraspinal abscess in the spinal cord. According to Garg and Somvanshi [5], the patient's neurological deficit might have been caused by *Tuberculosis* granuloma in extradural, intradural, or intramedullary regions, and an MRI imaging confirmed this on this patient.

Radiological examination is beneficial in finding extrapulmonary tuberculosis, especially in cases of TB spondylitis. Radiological examinations that can be used to support the diagnosis of TB spondylitis are X-rays, computed tomography scan (CT scan), and magnetic resonance imaging (MRI) [10,11]. The results of the chest X-ray in this case did not show the presence of tuberculosis. If the chest X-ray had shown TB's presence, the next stage would have been TB therapy.

This case used the thoracolumbar X-ray and MRI imaging methods. The thoracolumbar X-ray results confirmed a compression fracture in the thoracalis vertebral level 6. The MRI was used to see the results of the complete diagnosis. MRI is one of the most sensitive examinations for diagnosing TB spondylitis than X-ray and more [8] as well as higher accuracy [12] than CT scan. Salam and Rongpipi [13] describe the presentation of spondylitis using MRI are:

• T1: hypointense marrow in adjacent vertebrae
• T2: hyperintense marrow, disc, soft tissue infection
• T1 C+ (Gd): marrow, subligamentous, discal, dural enhancement.

In this case, an epidural abscess presentation was also found using the MRI imaging. Spinal epidural abscess is a severe pyogenic infection of the epidural space. The rapid accumulation of purulent material in the space between the dura matter and the osseoligamentous confines of the vertebral canal may injure the spine by direct compression or local ischemia [14]. Epidural abscess has a critical role in deciding whether a surgery is required or not [4].

MRI imaging of Epidural abscess in this case was presented as hypointense signals on T1-weighted and hyperintense signals on T2-weighted and enhancement at the margin. This is consistent with the characteristic findings of spinal epidural abscess on MRI, which include a high signal on T2-weighted imaging (T2WI) and a low signal on T1- weighted imaging (T1WI) [15,16].^.^

Other finding in this case was the presence of *Tuberculomas* or *tuberculous granulomas. Tuberculomas* or *tuberculous granulomas* are well-defined focal masses that result from Mycobacterium tuberculosis infection and are one of the more severe morphological forms of tuberculosis. MRI has become the diagnostic modality of choice for diagnosing spinal cord pathology. The intramedullary tuberculoma is well defined, circumscribed solitary or multiple, nodular or ring-enhancing lesions with prominent cord edema and swelling [17].

Usually, spinal tuberculosis may also have a tubercular focus anywhere else in the body, which significantly aids in diagnosing spinal tuberculoma [18]. Although *Tuberculomas* most commonly occur in the brain and the lung [19], in this particular case, spinal tuberculoma was found on accidental findings using MRI, presented as isointense signals on the T1-weighted with a ring shape enhancement of the intramedullary lesion at the L1 after contrast administration. This consistent with the presentation of tuberculoma using MRI, shown as the thickening of the spinal cord and oval lesions with a low T1-weighted image signal and a typical "target sign" T2-weighted image sign. After gadopentetate dimeglumine administration, the lesion's ring shape was enhanced, showing uneven wall thickness and sharp margins [20].

Based on the above findings, the patient was administered an antituberculosis drug therapy, and the surgery would be carried out after 3 months of treatment. The patient also confirmed that she had received antituberculosis therapy and undergone a good response, which further strengthened tuberculosis diagnosis enforcement. This intervention of this spondylitis tuberculosis management by administering antituberculosis therapy and surgical intervention is in line with Garg and Somvanshi [5] on their spinal tuberculosis treatment.

## Conclusion

The enforcement of the diagnosis in this patient was carried out with clinical findings, namely patient complaints supported by the results of the MRI contrast examination, namely a change in signal intensity in the vertebrae corpus. There was bone destruction due to the process of extracting mycobacterium tuberculosis germs and the presence of an abscess around the focus of infection.

Although clinical findings and MRI support the presence of spinal tuberculosis, to confirm the diagnosis, it is better to do a histopathological examination through tissue retrieval at the time of surgery [21].

## Patient consent

I, Puspita Permata Sari MD, declared that I have obtained patient's consent for this information relating to the subject matter above to appear in a journal article, or to be used for the purpose of a case report journal. The consent form document is ready and will be given to the journal should it be required.

## References

[1] Rajasekaran S, Soundararajan DCR, Shetty AP, Kanna RM (2018). Spinal tuberculosis: current concepts. Glob Spine J.

[2] de Jong BC, Antonio M, Gagneux S (2010). Mycobacterium africanum—review of an important cause of human tuberculosis in West Africa. PLoS Negl Trop Dis.

[3] Hange N, Somagutta MR, Sharma A, Agadi K, Ngaba NN, Paikkattil N (2022). Latent tuberculosis: challenges and opportunities for diagnosis and treatment. Discov Med.

[4] Vitriana V. Spondilitis tuberkulosa. BAGIAN ILMU KEDOKTERAN FISIK DAN REHABILITASI FK-UNPAD /RSUP.dr.HASAN SADIKIN FK-UI / RSUPN dr. CIPTOMANGUNKUSUMO; 2002.

[5] Garg RK, Somvanshi DS (2011). Spinal tuberculosis: a review. J Spinal Cord Med.

[6] Skoura E, Zumla A, Bomanji J (2015). Imaging in tuberculosis. Spec Issue Commem World Tuberc Day.

[7] Hasan Khan MN, Jamal AB, Hafeez A, Sadiq M, Rasool MU (2021). Is spinal tuberculosis changing with changing time?. Ann Med Surg.

[8] Garg D, Goyal V (2020). Spinal tuberculosis treatment: an enduring bone of contention. Ann Indian Acad Neurol.

[9] Lacerda C, Linhas R, Duarte R. (2017). Tuberculous spondylitis: a report of different clinical scenarios and literature update. Case Rep Med.

[10] Kusmiati T, Narendrani HP. (2016). POTT'S disease..

[11] Currie S, Galea-Soler S, Barron D, Chandramohan M, Groves C. (2011). MRI characteristics of tuberculous spondylitis. Clin Radiol.

[12] Ferrer MF, Torres LG, Ramírez OA, Zarzuelo MR, del Prado González N. (2012). Tuberculosis of the spine. A systematic review of case series. Int Orthop.

[13] Salam H, Rongpipi T. Tuberculous spondylitis. In: Radiopaedia.org [Internet]. Radiopaedia.org; 2010. Available from: https://radiopaedia.org/articles/8759

[14] Rosc-Bereza K, Arkuszewski M, Ciach-Wysocka E, Boczarska-Jedynak M. (2013). Spinal epidural abscess: common symptoms of an emergency condition: a case report. Neuroradiol J.

[15] Sharfman ZT, Gelfand Y, Shah P, Holtzman AJ, Mendelis JR, Kinon MD (2020). Spinal epidural abscess: a review of presentation, management, and medicolegal implications. Asian Spine J.

[16] Van Goethem JWM, van den Hauwe L, Parizel PM. (2008). Spinal imaging: diagnostic imaging of the spine and spinal cord. Am J Neuroradiol.

[17] Lalla R, Singh MK, Patil TB, Kumar N. (2013). MRI of the spinal tuberculoma, paravertebral tubercular abscess and pulmonary tuberculosis. BMJ Case Rep.

[18] DeSanto J, Ross JS. (2011). Spine infection/inflammation. Radiol Clin.

[19] Smith D, Stanislavsky A. Tuberculoma. Radiopaedia.org. 2013;

[20] Lu M. (2010). Imaging diagnosis of spinal intramedullary tuberculoma: case reports and literature review. J Spinal Cord Med.

[21] Lee CM, Lee Y, Kang SJ, Kang CK, Choe PG, Song KH (2022). Positivity rates of mycobacterial culture in patients with tuberculous spondylitis according to methods and sites of biopsies: an analysis of 206 cases. Int J Infect Dis.

